# Renal Resistive Index from Renal Hemodynamics to Cardiovascular Risk: Diagnostic, Prognostic, and Therapeutic Implications

**DOI:** 10.3390/diseases13060178

**Published:** 2025-06-09

**Authors:** Giulio Geraci, Pietro Ferrara, Luigi La Via, Alessandra Sorce, Vincenzo Calabrese, Giuseppe Cuttone, Valentina Paternò, Francesco Pallotti, Gianluca Sambataro, Luca Zanoli, Jacob George, Riccardo Polosa, Giuseppe Mulè, Caterina Carollo

**Affiliations:** 1 Department of Medicine and Surgery, “Kore” University of Enna, 94100 Enna, Italy; giulio.geraci@unikore.it (G.G.); vincenzo.calabrese@unikore.it (V.C.); giuseppe.cuttone@hotmail.it (G.C.); francesco.pallotti@unikore.it (F.P.); gianlucasambataro@unikore.it (G.S.); polosa@unict.it (R.P.); 2 Center for Public Health Research, University of Milan–Bicocca, 20900 Monza, Italy; pietro.ferrara@unimib.it; 3 Laboratory of Public Health, IRCCS Istituto Auxologico Italiano, 20149 Milan, Italy; 4 Department of Anesthesia and Intensive Care 1, University Hospital Policlinico “G. Rodolico-San Marco”, 95123 Catania, Italy; 5 Department of Health Promotion, Mother and Child Care, Internal Medicine and Medical Specialties, Unit of Nephrology and Dialysis, School of Medicine, University of Palermo, 90100 Palermo, Italy; alessandra.sorce@community.unipa.it (A.S.); giuseppe.mule@unipa.it (G.M.); caterina.carollo@unipa.it (C.C.); 6 Unit of Internal Medicine, “Umberto I” Hospital of Enna, 94100 Enna, Italy; valentinapaterno91@gmail.com; 7 Department of Clinical and Experimental Medicine, University of Catania, 95100 Catania, Italy; luca.zanoli@unict.it; 8 Ninewells Hospital, School of Medicine, University of Dundee, Dundee DD1 9SY, Scotland, UK; j.george@dundee.ac.uk; 9 Centre of Excellence for the Acceleration of HArm Reduction (CoEHAR), University of Catania, 95100 Catania, Italy; 10 European Society of Hypertension Excellence Center, Chair of Nephrology, University of Palermo, 90100 Palermo, Italy

**Keywords:** renal resistive index, cardiovascular risk, arterial stiffness, renal hemodynamic, hypertension, Doppler ultrasonography

## Abstract

Duplex-Doppler ultrasonography has become an essential tool in the diagnosis and management of kidney diseases, allowing clinicians to assess renal hemodynamics, detect vascular abnormalities, and monitor disease progression. Among the various Doppler-derived parameters, the renal resistive index (RRI) has gained particular attention both as a diagnostic tool and a prognostic marker in nephrology. Traditionally considered an indicator of parenchymal perfusion, recent evidence highlights its strong association with systemic hemodynamic factors, particularly arterial stiffness, positioning RRI as a valuable tool for evaluating patients with systemic vascular impairment, such as hypertension, diabetes mellitus, and atherosclerosis. RRI has been strongly linked to vascular damage, which in turn is influenced by inflammation and endothelial dysfunction, making it a reliable marker of cardiovascular damage and a potential predictor of cardiovascular risk. Furthermore, emerging studies suggest that RRI could serve as a dynamic parameter to monitor vascular changes induced by therapeutic interventions. This narrative review summarizes the classic and evolving applications of RRI, from its origin as a renal hemodynamic marker to its emerging role as a systemic vascular biomarker with diagnostic and prognostic significance in cardiovascular and metabolic diseases.

## 1. Introduction

Since the early 1970s, several authors have highlighted the role of Duplex Doppler ultrasonography in the diagnosis and management of kidney diseases, leading to significant advancements in the non-invasive assessment of renal function and hemodynamics [1,2]. This imaging technique has been widely applied to evaluate blood flow characteristics in renal arteries and microcirculation, allowing clinicians to identify vascular abnormalities and monitor disease progression. Over the years, numerous Doppler-derived parameters have been investigated in various renal pathologies to assess intrarenal perfusion and resistance [1,2,3]. Among these, a Doppler-derived index, introduced by Pourcelot in 1974, demonstrated a strong correlation with the renal vascular resistance and was therefore named the renal resistive index (RRI) [1]. This index—calculated using the formula RRI = (peak systolic velocity—end diastolic velocity)/peak systolic velocity—was initially employed both as a diagnostic tool and a prognostic marker in kidney diseases.

Elevated RRI values were traditionally interpreted as an indication of increased intrarenal impedance, reflecting structural and functional alterations in renal microvasculature. As a result, RRI became a widely utilized parameter in nephrology, with studies supporting its role in differentiating between obstructive and non-obstructive uropathy, predicting the response to treatment, and monitoring renal allograft function in kidney transplant recipients. It was particularly useful in detecting renovascular disease and chronic kidney disease (CKD), where increased intrarenal vascular resistance is a hallmark of disease progression [2].

However, more recent evidence suggests that RRI is not solely a marker of parenchymal perfusion but is also influenced by systemic hemodynamic factors, particularly arterial stiffness, and overall vascular compliance [3]. Studies have demonstrated that systemic vascular changes, including alterations in central blood pressure, pulse wave velocity, and cardiovascular aging, exert a significant impact on RRI values [2]. This broader perspective has led to a shift in the clinical interpretation of RRI, moving beyond its traditional use in assessing renal pathology to recognizing its potential as a global vascular marker.

Systemic vascular changes appear to have a greater impact on RRI than intrarenal resistance [2]. Consequently, RRI is now being investigated as a key parameter in evaluating individuals with systemic vascular impairment, including those with hypertension, diabetes mellitus, and atherosclerosis. Given its association with arterial stiffness and systemic vascular damage, RRI has emerged as a potential predictor of overall cardiovascular risk. Elevated RRI values have been linked to an increased risk of cardiovascular events, organ damage, and mortality, further emphasizing the need for a more integrated approach when interpreting this parameter in clinical practice [2].

RRI has, therefore, evolved from being merely a renal Doppler index to a more comprehensive vascular biomarker with implications beyond nephrology. Its potential role in cardiovascular risk stratification highlights the importance of further research to refine its clinical applications and establish standardized reference values in different patient populations.

Although this is a narrative review, we aimed to provide a comprehensive and up-to-date synthesis of the literature. We conducted a non-systematic search of the PubMed and Scopus databases up to March 2025 using combinations of the following keywords: “renal resistive index” OR “RRI” AND “Doppler”, “vascular resistance”, “arterial stiffness”, “cardiovascular risk”, “chronic kidney disease”, “hypertension”, “diabetes”, and “critical care”. We included original research articles, meta-analyses, and relevant reviews published in English involving human subjects. Priority was given to clinical studies that investigated the diagnostic, prognostic, or therapeutic implications of RRI across various patient populations. Reference lists of key articles were also screened to identify additional relevant studies.

## 2. Renal Vascular Resistance: Pathophysiology and Renal Diseases

Following the earliest evidence, in vitro studies confirmed that RRI directly correlates with downstream intrarenal vascular resistance [4,5]. Similar findings were observed in in vivo animal models, where increased intrarenal impedance was associated with elevated RRI values [6]. Subsequent studies by Bude et al. reinforced these observations, demonstrating that in the presence of vascular compliance, RRI remained dependent on downstream vascular resistance and increased proportionally with rising overall impedance [3].

Further experimental validation was provided by monitoring RRI values of rabbit kidneys subjected to pulsatile perfusion while increasing renal pelvic pressure via the ureter. The graded elevation in renal pelvic pressures led to a corresponding rise in renal vascular resistance, which was reflected by higher RRI values [7]. In human studies, Platt et al. assessed 21 kidneys (14 with obstruction and 7 without obstruction) immediately before a percutaneous nephrostomy. Their findings revealed that 13 of the 14 obstructed kidneys exhibited RRI values >0.70, whereas none of the non-obstructed kidneys had RRI values exceeding this threshold. Importantly, the resolution of the obstruction through percutaneous nephrostomy led to a marked reduction in RRI values [8]. These observations were further corroborated in renal transplant recipients, where higher RRI values were observed only in cases where urinary dilatation had an obstructive origin [9].

Changes in vascular resistance appear to be linked to the increased interstitial pressure, which exerts mechanical stress on the wall of intraparenchymal vessels. This leads to an increase in transmural pressure, ultimately resulting in higher RRI values. It is plausible that various pathological conditions characterized by increased interstitial pressure, such as interstitial fibrosis or extrinsic compression, operate through a similar mechanism, further reinforcing the diagnostic significance of RRI in these contexts [10]. Supporting this hypothesis, several studies have established correlations between renal hemodynamics and histopathological findings of tubular necrosis and interstitial fibrosis [11,12]. However, the sensitivity and specificity of RRI vary depending on the degree of obstruction, likely due to its differential impact on the transmural pressure [13]. For instance, in two studies on patients with renal colic, an RRI threshold of >0.70 was found to be fairly sensitive and specific for diagnosing acute obstruction [14]. Contrarily, subsequent studies reported significantly lower sensitivity (44%) and specificity (82%) [15], likely due to variations in obstruction severity, the vasodilatory effects of nonsteroidal anti-inflammatory drugs, and differences in patient hydration status.

Another mechanism contributing to increased RRI is the reduction in cross-sectional area of the distal arterial bed. This hypothesis has been supported by histopathological studies conducted by Bigè et al., which demonstrated a correlation between RRI values >0.65 and severe renal arteriosclerosis [11]. In line with the aforementioned evidence, elevated RRI values have also been observed in non-obstructive renal parenchymal conditions that primarily affect the vasculature or tubulo-interstitium (e.g., acute tubular necrosis, interstitial nephritis, systemic lupus erythematosus, cryoglobulinemia and other vasculitides). In contrast, primarily glomerular diseases are generally not associated with significant changes in RRI [16].

Numerous studies have highlighted the role of RRI in the diagnosis and management of renal artery stenosis and renovascular disease. In a cohort of 5950 hypertensive patients, Radermacher et al. demonstrated that RRI played a crucial role in guiding treatment decisions for individuals with renal artery stenosis. Specifically, an RRI ≥ 0.80 reliably identified patients who were unlikely to benefit from angioplasty or surgery, as these interventions did not lead to improvements in renal function, blood pressure control, or kidney survival [17]. However, it has limited utility in differentiating between the most common causes of transplant dysfunction, such as acute tubular necrosis, rejection, and immunosuppression toxicity [13,18].

This diagram summarizes the multifactorial mechanisms contributing to elevated RRI. It highlights the influence of intrarenal vascular resistance, interstitial pressure, and microcirculatory damage, as well as the impact of systemic hemodynamic factors such as arterial stiffness, pulse pressure, and central blood pressure. The figure also outlines the role of endothelial dysfunction, inflammation, and atherosclerosis and emphasizes the bidirectional relationship between renal and systemic vascular damage.

In patients with CKD, a strong correlation has been observed between renal hemodynamic indices, particularly RRI and pulsatility index, and the severity of renal impairment [19]. Hanamura et al. further described higher RRI values in advanced CKD stages [20] and demonstrated that an RRI > 0.75 was associated with a more rapid decline in renal function in CKD patients.

In a cohort of 162 patients newly diagnosed with renal disease, with or without renal artery stenosis, Radermacher et al. identified an RRI ≥ 0.80 as an independent factor of disease progression over a mean follow-up of 3 ± 1.4 years [21]. These findings were later corroborated in studies utilizing a lower RRI cut-off, further emphasizing the prognostic value of this parameter [22]. Large-scale studies [11,12] have also established that RRI correlates with histopathological features of advanced CKD, irrespective of etiology. However, data regarding early stages of CKD remain inconsistent, with some conflicting findings in the literature.

**Figure 1:**
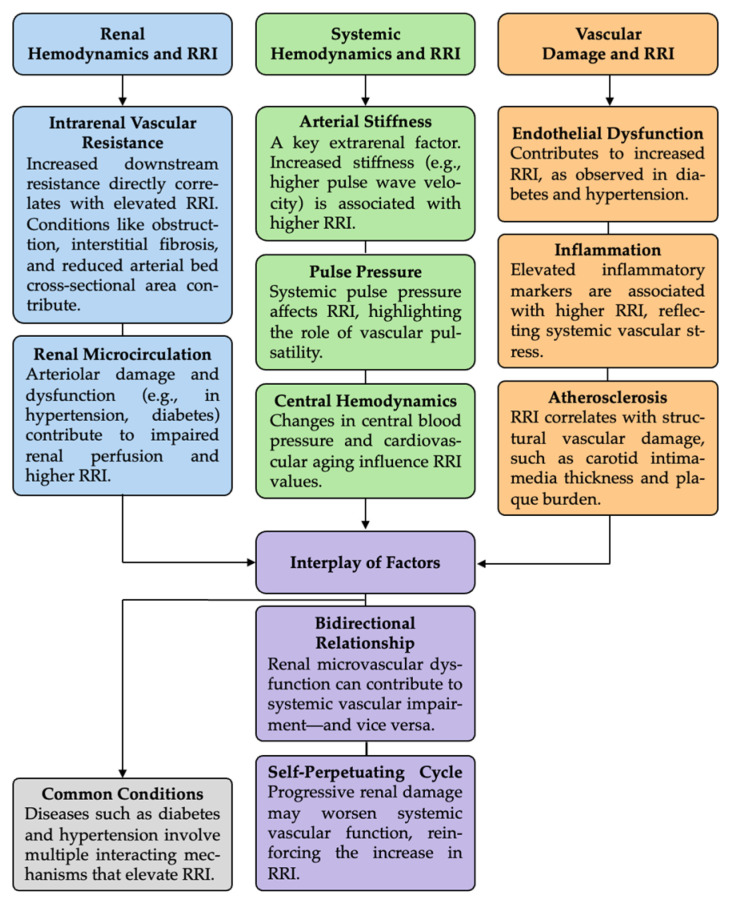
Schematic overview of the pathophysiological determinants of the renal resistive index (RRI).

A visual synthesis of the multifactorial pathophysiology of RRI is presented in Figure 1, illustrating its relevance across various contexts.

## 3. Renal Resistive Index in Diabetes Mellitus

Following the initial evidence establishing the association between RRI and renal vascular resistance, several authors have proposed that extrarenal factors may also influence this index. Early observations by Saunders et al. indicated that RRI in the uterus was affected not only by downstream vascular resistance but also by other factors related to blood pulsatility [23]. These early findings were subsequently confirmed by an ex vivo study conducted by Tublin et al., in which rabbit kidneys were perfused by a pulsatile system. Their results further supported the notion that RRI is strongly influenced by pulse pressure, reinforcing the concept that systemic hemodynamic factors play a crucial role in determining RRI values [24].

It thus became evident that RRI is influenced by multiple upstream factors beyond mere renal vascular resistance [25]. Consequently, many authors shifted their focus to explore the potential association between RRI and systemic vascular alterations, particularly in conditions such as hypertension and diabetes.

In a study involving 245 patients with or without renal impairment, Otha et al. observed that RRI values in both the main renal arteries and interlobar arteries were significantly higher in patients with diabetic nephropathy compared to those with chronic glomerulonephritis, nephrosclerosis, or no kidney disease [26]. These differences persisted even after adjusting for multiple variables, including creatinine clearance. Beyond its role in CKD progression, RRI has been shown to predict early development and progression of renal involvement in diabetes. An elevated RRI may indicate underlying renal damage in diabetic individuals, including ischemic nephropathy associated with endothelial dysfunction, a hallmark of diabetic vasculopathy. Although RRI serves as a useful indicator of nephropathy in diabetic patients, even in the absence of microalbuminuria, Afsar et al. have reported that higher RRI values were found in diabetic patients with reduced creatinine clearance and increased 24 h urinary albumin excretion compared to those with normal creatinine clearance and albumin levels [27]. Additionally, multiple studies have demonstrated that higher RRI values are strongly associated with macroalbuminuria, particularly in diabetic patients, even after adjusting for glomerular filtration rate (GFR) [28,29].

In addition to these findings, Boeri et al. reported that diabetic individuals exhibited significantly higher RRI values compared to controls, attributing this difference to intrarenal vascular alterations [30]. More recently, Kawai et al. found that patients with diabetes mellitus had a significantly higher RRI than those without diabetes, despite the absence of statistically significant differences in eGFR between the two groups. This suggests that extrarenal factors contribute to increased RRI values, in particular pulse wave velocity [31].

## 4. Renal Resistive Index in Arterial Hypertension and Cardiovascular Diseases

Arterial hypertension, a major contributor to end-organ damage, has shown a consistent relationship with increased RRI, underscoring the impact of chronic pressure overload on renal microcirculation and systemic vascular integrity. As early as 1995, Veglio et al. demonstrated that both the duration and severity of hypertension correlate with higher RRI, primarily due to increased vascular impedance from renal arteriolar damage [32]. This may result from two concomitant mechanisms: (i) an increase in renal vascular tone, secondary to sustained hypertension; and (ii) the presence of intrarenal arteriolosclerosis, which progressively alters renal microcirculation. While early changes may be reversible, long-term alterations often lead to irreversible nephrosclerosis. Notably, Ruilope et al. suggested that renal microvascular dysfunction may not only result from hypertension but also contribute to its development and persistence [33].

These findings have sparked growing interest in the association between RRI and early organ damage in individuals at cardiovascular risk, especially in hypertensive patients. In a study involving 211 untreated patients with essential hypertension, Pontremoli et al. [34] observed a positive correlation between RRI, carotid intima-media thickness (cIMT), and other markers of early organ damage. Similar associations have since been confirmed by other authors [35]. RRI has also been linked to cIMT—a well-established marker of vascular damage—even in patients with impaired renal function. In a study of 140 CKD patients, Heine et al. found that RRI was significantly correlated with the type and stage of kidney disease, as well as with systemic atherosclerotic changes, which were assessed using ankle–brachial index (ABI) and cIMT [36]. Ishimura et al. further supported this by showing a relationship between RRI and carotid IMT in diabetic nephropathy, suggesting RRI’s value in detecting subclinical vascular disease [37].

Geraci et al. highlighted that in hypertensive patients, with or without renal impairment, RRI strongly correlates with the severity of carotid atherosclerosis (cIMT and plaque burden) [38,39]. The relationship may be mediated by two concomitant mechanisms. One possible link is arterial stiffness, a well-documented determinant of vascular aging and atherosclerotic progression. Supporting this, several studies, including one by Otha et al. involving 245 subjects, have demonstrated a significant univariate correlation between RRI and measures of arterial stiffness such as brachial–ankle pulse wave velocity (baPWV) [26,40]. Our group recently obtained similar results using the aortic PWV, a more direct measure of central arterial stiffness compared to baPWV. Comparable findings were also reported by Hashimoto and Ito, who observed a strong correlation between aPWV and RRI in 133 hypertensive patients [41,42]. Increased arterial stiffness is thought to expose the renal circulation to greater hemodynamic stress, where pulse pressure plays a more significant role than mean arterial pressure. This increased pulsatile load from the central aorta down to the renal microvasculature may result in higher renal vascular resistance and consequently elevated RRI values [43]. On the other hand, RRI itself is a marker of renal pulsatile flow dynamics. This interaction suggests the presence of a self-perpetuating cycle, where renal impairment exacerbates systemic vascular dysfunction, which further amplifies renal microvascular resistance [44].

Beyond arterial stiffness, the association between RRI and carotid atherosclerosis may also involve arterial calcifications. In 77 CKD patients, Stefan et al. reported significant correlations between RRI and abdominal aortic calcifications, as well as cIMT [45]. RRI has also been linked to endothelial dysfunction and inflammation—key drivers of atherosclerosis [46]. In 61 hypertensive patients, RRI was associated with inflammatory markers, particularly with C-reactive protein (CRP) levels [47]. Furthermore, experimental studies showed that renal nitric oxide synthase inhibition—which reduces nitric oxide levels—results in a marked increase in RRI [48]. These findings highlight the contribution of inflammation, nitric oxide deficiency, and endothelial dysfunction to elevated RRI, reinforcing its value as a systemic vascular marker.

Several studies have linked RRI to markers of target organ damage, particularly in hypertension. Alterini et al. found significantly higher RRI values in 28 hypertensive patients compared to 13 normotensive controls, particularly among those with increased left ventricular mass, suggesting a strong link between RRI and left ventricular hypertrophy (LVH) [49]. As an expression of arterial impedance, RRI correlated with the presence and severity of LVH, reinforcing its role as a marker of hypertensive heart disease. Tedesco et al. later confirmed, among 566 enrolled individuals, that hypertensive patients with an RRI ≥ 0.70 exhibited increased cIMT and left ventricular mass index (LVMI), the latter associated with subclinical impairment of left ventricle diastolic function [50]. These patients also showed a higher prevalence of LVH, carotid plaques, and microalbuminuria compared to those with RRI < 0.70 [50]. These findings were further corroborated by Pontremoli et al., who reported similar associations [34].

Consistent with its multifactorial nature, studies have demonstrated that RRI is influenced by age and pulse pressure [38,51] and associated with greater systemic 24 h blood pressure load, abnormal circadian pattern patterns (i.e., non-dipping profiles), sympathetic activity, and elevated uric acid [52]. Recent work by our group further demonstrated a significant association between RRI, retinal damage, and choroidal thickness in hypertensive patients—with or without diabetes—regardless of renal function. These findings provide further evidence that RRI serves as an early marker of systemic vascular changes, even in the absence of overt cardiovascular disease [53,54].

Moreover, some evidence suggests that the RRI may also serve as a valuable non-invasive marker in patients with coronary artery disease (CAD). In a cohort of 130 hypertensive patients referred for elective coronary angiography, the authors found that higher RRI values were associated with coronary atherosclerotic burden, as measured by the Gensini Score. This association remained significant after adjustment for age and left ventricular ejection fraction [55]. These findings support the hypothesis that renal vascular dysfunction, as reflected in Doppler-derived indices, may mirror early coronary vascular damage and provide insight into systemic atherosclerotic involvement in hypertensive patients.

Lastly, RRI has also emerged as a potential prognostic marker in patients with heart failure, particularly in those with heart failure with preserved ejection fraction (HFpEF). In a study involving 90 HFpEF patients and 90 age- and sex-matched hypertensive controls without heart failure, Ennezat et al. demonstrated that mean RRI was significantly higher in the HFpEF group. Notably, this difference was observed despite comparable or even lower blood pressure levels in HFpEF patients, suggesting that the elevated RRI reflected intrinsic intrarenal vascular dysfunction rather than simply systemic hemodynamics. Multivariable analysis confirmed that RRI was independently associated with HFpEF and served as a significant predictor of adverse outcomes, including all-cause mortality and heart failure hospitalization [56].

## 5. Prognostic Value of Renal Resistive Index

Although numerous studies have demonstrated a strong association between RRI and vascular damage, only recently have some authors highlighted the potential prognostic significance of renal hemodynamic alterations in predicting renal and cardiovascular outcomes in hypertensive patients as well as in other patient populations. In a study on older patients, Pearce et al. found that over a median follow-up of 7 years, higher RRI values were associated with increased cardiovascular morbidity and mortality [57]. More recently, in a cohort of 426 patients with primary hypertension and no prior cardiovascular diseases, Doi et al. investigated the prognostic role of RRI over a mean follow-up of 3.1 years. Their findings demonstrated that RRI was an independent predictor of worse renal and cardiovascular outcomes, particularly when combined with reduced eGFR. Even after adjusting for multiple confounders, RRI remained a significant cardiovascular prognostic factor, further supporting its clinical relevance in risk stratification [58].

The prognostic role of RRI on cardiovascular mortality and renal events has also been evaluated in 1962 patients with CKD without renal artery stenosis. Over a median follow-up of 2.2 years, RRI ≥ 0.70 exhibited a significantly higher mortality risk, even after adjusting for multiple covariates. The authors concluded that RRI assessment “may allow early identification of those who are at risk, thereby potentially preventing or delaying adverse outcomes” [59]. Similarly, in a retrospective study of 1685 renal transplant recipients, both with and without diabetes, RRI changes demonstrated a strong association with overall mortality, irrespective of renal function [60].

RRI is increasingly recognized as a systemic cardiovascular prognostic marker, rather than solely a renal parameter. A striking example of this is the predictive role of RRI in all-cause mortality among patients with systemic sclerosis, regardless of renal function [61]. Further supporting this perspective, the J-VAS study, conducted on 1598 patients with atherosclerotic cardiovascular disease (ASCVD) and followed for 1.9 years, found that RRI was significantly associated with an increased risk of cardiovascular events in these patients [62]. Collectively, this evidence underscores the growing recognition of RRI as a robust, non-invasive marker with independent prognostic value for adverse renal and cardiovascular outcomes, supporting its potential role in risk stratification across diverse patient populations.

Beyond its cross-sectional prognostic value, RRI may serve as a dynamic marker to track vascular and renal disease progression. Research has demonstrated that longitudinal increases in RRI are associated with worsening renal function, higher cardiovascular risk, and adverse clinical outcomes in patients with CKD, hypertension, or diabetes mellitus [57]. For example, follow-up assessments of RRI in patients treated with renin-angiotensin system inhibitors or SGLT2 inhibitors have shown that a reduction in RRI may parallel improvements in vascular compliance and microvascular health [63]. These findings suggest that temporal trends in RRI could reflect treatment efficacy or early disease acceleration, warranting further exploration in interventional and real-world cohorts.

## 6. Renal Resistive Index in Acute Critical Setting: Opportunities and Challenges

While the RRI has demonstrated significant diagnostic and prognostic value across a range of clinical settings, its integration into routine practice presents both promising opportunities and notable challenges. Due to its potential to provide valuable insights into renal function and hemodynamics, RRI has gained increasing attention in critically ill patients, particularly within intensive care unit (ICU) settings, where renal perfusion is frequently compromised by systemic hemodynamic instability, sepsis, and the use of vasoactive drugs. Monitoring RRI in these patients offers a non-invasive method for assessing renal hemodynamics and detecting early signs of kidney injury, a common complication in critically ill patients that often leads to increased morbidity and mortality.

Several studies have shown that an elevated RRI correlates with the onset of acute kidney injury (AKI) in ICU patients. In a study by Schnell et al., ICU patients with an RRI ≥ 0.8 had a higher risk of developing AKI compared to those with a lower RRI [64]. Notably, RRI serves as an early indicator of renal injury, potentially before changes in serum and urine cystatin C levels and serum creatinine levels occur. Similarly, in a cohort of 125 ICU patients diagnosed with AKI, high RRI values were independently associated with persistent AKI at ICU discharge and increased in-ICU mortality [65]. Beyond AKI detection, the prognostic value of RRI extends to overall survival and long-term renal outcomes in critically ill patients. A study by Darmon et al. demonstrated that elevated RRI values in septic patients were associated with higher ICU mortality and worse renal outcomes [66]. Similarly, Lerolle et al. observed comparable results in a prospective clinical study on patients with septic shock [67].

Although there is a correlation between intrarenal hemodynamics and the risk of future renal and cardiovascular events, vasoactive medications, such as norepinephrine, are frequently used in ICU patients and can significantly affect renal blood flow and, consequently RRI values. Therefore, it is essential to consider the influence of these medications when interpreting RRI measurements in critically ill patients.

In a study by Deruddre et al., norepinephrine was titrated over two hours in 11 patients with septic shock to achieve a mean arterial pressure (MAP) above 65 mmHg. When MAP increased from 65 to 75 mmHg, urinary output significantly improved, and RRI decreased from 0.75 to 0.70. However, no further changes were observed when MAP was increased from 75 to 85 mmHg, highlighting the complex relationship between RRI, renal hemodynamics, and AKI in ICU setting [68]. For this reason, several limitations must be considered when using RRI in critically ill patients. These include the influence of blood pressure and cardiac output [69], interobserver variability, and the lack of standardized diagnostic or prognostic thresholds. Nevertheless, an RRI value > 0.70 is commonly considered abnormal [70]. It is worth noting that there is a need to interpret RRI dynamics within the broader context of pharmacologic intervention, considering both the therapeutic goal and potential renal side effects. Incorporating RRI monitoring into protocols for vasoactive drug titration may offer a non-invasive adjunct to optimize perfusion strategies while minimizing renal injury risk. Lastly, future efforts should aim to improve the standardization and reproducibility of RRI measurement in critically ill patients. Structured operator training and adherence to predefined scanning protocols may reduce interobserver variability, especially when RRI is used as a monitoring tool in an ICU settings. Furthermore, emerging technologies—including automated Doppler waveform analysis and artificial intelligence–based ultrasound software—may enable more objective, real-time assessment of RRI with minimal operator dependency [71].

## 7. Renal Resistive Index in Pediatric Nephrology

Although RRI has been extensively studied in adult populations, its application in pediatric nephrology is still evolving. In children, RRI is gaining interest as a non-invasive tool to assess renal hemodynamics in various clinical settings, including congenital anomalies of the kidney and urinary tract, pediatric hypertension, and renal transplantation. Studies showed that RRI can help distinguish between obstructive and non-obstructive uropathies in neonates and young children, particularly in cases of hydronephrosis. In this context, elevated RRI values have been associated with increased intrarenal pressure and impaired urinary drainage. However, age-related physiological variations must be considered when interpreting RRI in pediatric patients [72,73,74,75].

In pediatric hypertension, increased RRI has been linked to early vascular alterations and subclinical organ damage. Cilsal and Koc evaluated RRI in 75 children, including 48 with newly diagnosed hypertension and 27 healthy controls. RRI was significantly higher in hypertensive children and positively correlated with systolic blood pressure, pulse pressure, and LVMI. Pulse pressure and LVMI were identified as independent predictors of RRI, supporting its role as a marker of early hemodynamic and structural alterations in pediatric hypertension [74].

RRI has also been used to monitor renal allograft perfusion in pediatric kidney transplant recipients [76]. Elevated post-transplant RRI values may indicate acute rejection, vascular complications, or chronic allograft dysfunction. However, interpreting RRI in this setting remains challenging due to the influence of extrarenal factors such as systemic blood pressure, hydration status, and immunosuppressive regimens.

A cross-sectional study by Swarnim et al. evaluated RRI in 50 children and adolescents aged 6–18 years with nephrotic syndrome, comparing steroid-sensitive (SSNS) and steroid-resistant (SRNS) patients. RRI values were significantly higher in SRNS patients, particularly at the mid-pole of both kidneys. Children with focal segmental glomerulosclerosis (FSGS) also showed higher RRI values than those with minimal change disease. Additionally, prolonged cyclosporine use (>2 years) was associated with elevated RRI, suggesting its potential as a non-invasive marker of chronic tubulointerstitial damage in this population [72].

A recent prospective observational study by Rajangam et al. assessed the predictive utility of RRI measured via point-of-care ultrasound for sepsis-associated acute kidney injury (AKI) in 79 critically ill children aged 1–12 years. Children who developed AKI by day 3 had significantly higher mean RRI values than those who did not (0.72 vs. 0.65). ROC analysis showed that in children aged 1–4 years, an RRI ≥ 0.71 predicted AKI with 100% sensitivity and 46.2% specificity, while in children aged 5–12 years, an RRI ≥ 0.69 had 70% sensitivity and 77.5% specificity. RRI and eGFR were independent predictors of AKI in multivariate analysis. These findings suggest that RRI may be a useful early marker of AKI in pediatric sepsis, although its standalone predictive power remains limited [73].

Despite these promising findings, the use of RRI in pediatrics is still limited by the lack of age-specific reference values and the variability introduced by growth and maturation. Most available data come from small, single-center studies. Further research is needed to define standardized protocols, validate diagnostic thresholds, and clarify the prognostic value of RRI in pediatric kidney diseases.

## 8. Future Directions

Although there is strong evidence supporting the association between RRI, subclinical vascular damage, renal dysfunction and cardiovascular mortality, there remains a lack of studies definitively demonstrating whether RRI is influenced by chronic cardiovascular therapies and whether modifications in RRI translate into actual change in renal and cardiovascular outcomes. In this regard, a recent prospective study on diabetic patients found that RRI decreased after 26 weeks of treatment with glucagon-like peptide 1 receptor agonists (GLP-1 RAs) and sodium–glucose cotransporter 2 (SGLT2) inhibitors [63]. These drugs have been shown to reduce cardiovascular and renal events in diabetic patients, reinforcing the potential clinical relevance of RRI monitoring. Notably, in the study, the improvement in RRI was independent of age, sex, diabetes duration, changes in body mass index, waist circumference, glycosylated hemoglobin, and eGFR.

In addition to cardiometabolic agents, the impact of vasoactive drugs—commonly used in critical care—on RRI values warrants careful consideration. Vasopressors such as norepinephrine can acutely modify renal perfusion [77], leading to transient changes in RRI that may not reflect structural renal injury. Studies have shown that titration of norepinephrine to improve mean arterial pressure can lower RRI and increase urine output, although this effect may plateau at higher pressure thresholds [68]. These findings suggest that RRI is not only influenced by chronic vascular remodeling but also by acute hemodynamic shifts, raising important considerations for interpretation in the ICU setting.

This finding highlights the potential role of RRI as a tool for detecting early kidney damage and monitoring disease progression during treatment. Looking ahead, it is conceivable that RRI could be used to track vascular changes induced by cardiovascular and metabolic therapies, allowing for real-time monitoring of treatment efficacy. This could eventually lead to personalized therapeutic strategies, where treatment adjustments are guided by RRI variations to optimize renal and cardiovascular outcomes. Moreover, future studies should investigate whether changes in RRI following specific therapies reflect reversible functional adaptations or permanent microvascular damage. Longitudinal studies and randomized trials are needed to assess whether RRI variation predicts long-term outcomes or treatment response and to clarify potential adverse effects (e.g., renal hypoperfusion) in specific subgroups. In this context, prospective interventional trials are essential to validate the role of RRI as a dynamic biomarker of therapeutic response. Specifically, randomized controlled trials (RCTs) should explore whether reductions in RRI following treatment with cardiometabolic agents (e.g., SGLT2 inhibitors, GLP-1 receptor agonists, antihypertensives) translate into improved renal or cardiovascular outcomes.

Furthermore, future studies should aim to integrate RRI assessment with molecular biomarkers of vascular health, including markers of endothelial function (e.g., nitric oxide availability, flow-mediated dilation), inflammation (e.g., C-reactive protein, interleukins), and fibrosis (e.g., TGF-β1, galectin-3). This integrated approach may provide mechanistic insights into the pathways modulating RRI and help identify patients at risk of treatment resistance. Ultimately, coupling Doppler-based RRI monitoring with molecular profiling could support more precise and personalized therapeutic strategies, enabling clinicians to dynamically adjust treatment based on real-time vascular response.

Looking ahead, RRI could be incorporated into therapeutic decision-making frameworks, complementing established markers such as eGFR, albuminuria, and blood pressure control. For example, in patients with hypertension or diabetes, serial RRI assessments could help identify individuals with subclinical microvascular damage who may benefit from earlier initiation or intensification of cardioprotective therapies (e.g., RAS blockers, SGLT2 inhibitors, GLP-1RAs). In CKD, RRI might aid in distinguishing patients with predominant vascular or tubulointerstitial pathology, potentially guiding choices between anti-inflammatory, antifibrotic, or hemodynamic treatment strategies.

We propose a conceptual framework in which RRI is integrated into personalized treatment algorithms as a modifiable marker of vascular health, used in tandem with other parameters for longitudinal monitoring. Additionally, with further validation, RRI could be incorporated into electronic health record-based clinical decision-support systems or multivariable risk scores, improving risk stratification and treatment individualization in both outpatient and critical care settings.

## 9. Conclusions

**Figure 2:**
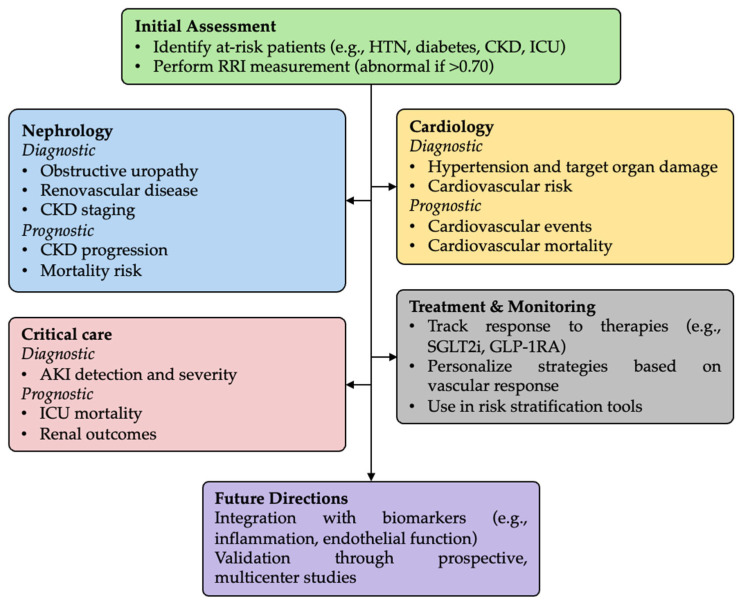
Clinical framework and decision algorithm for the use of renal resistive index (RRI) in clinical practice.

The clinical utility of RRI in patient stratification and treatment monitoring is illustrated in Figure 2, which also highlights its integration into future diagnostic and decision-making pathways.

This figure outlines a structured approach to the clinical use of RRI in various settings. It includes initial patient assessment, nephrology, cardiology, and critical care applications—each with diagnostic and prognostic relevance. The framework also describes the use of RRI in treatment monitoring, personalized medicine, and risk stratification, and proposes future directions, including integration with biomarkers and validation in prospective studies.

The extensive body of evidence presented highlights the evolution of RRI from a renal hemodynamic parameter to a systemic cardiovascular marker with significant prognostic implications. Initially recognized as a reflection of renal vascular resistance, RRI has since been shown to be strongly influenced by systemic hemodynamic factors, including arterial stiffness, endothelial dysfunction, and inflammation. Its close association with carotid atherosclerosis, left ventricular hypertrophy, and abnormal circadian blood pressure patterns further supports its role in assessing early organ damage in individuals with cardiovascular risk. Moreover, recent studies underscore the prognostic value of RRI in predicting adverse renal and cardiovascular outcomes. Elevated RRI has been linked to higher cardiovascular morbidity and mortality in hypertensive patients, CKD populations, and renal transplant recipients, reinforcing its potential utility in risk stratification. Looking forward, RRI may become a key parameter in personalized medicine, aiding in real-time monitoring of disease progression and therapeutic responses in cardiovascular and metabolic diseases.

Beyond its diagnostic applications, RRI has demonstrated prognostic value in predicting renal function decline, cardiovascular events, and overall mortality. Its relevance has expanded into critical care settings, where it serves as a non-invasive tool for assessing renal hemodynamics and detecting early kidney injury in critically ill patients. However, several challenges remain, including the influence of vasoactive medications, interobserver variability, and the lack of standardized thresholds for clinical decision-making.

As research continues to uncover the multifactorial nature of RRI, its clinical applications are expanding beyond nephrology to encompass cardiovascular medicine and global risk assessment. However, the current evidence is largely derived from retrospective or single-center studies, which may limit generalizability. Future prospective and multicenter investigations, including diverse age groups, comorbidities, and ethnic backgrounds, are crucial to validate RRI thresholds and ensure broader clinical applicability. Ultimately, RRI represents a promising tool for early vascular risk assessment, with the potential to improve long-term patient outcomes through timely intervention and prevention strategies.

## Data Availability

No new data were generated in this research.

## Abbreviations

The following abbreviations are used in this manuscript:

ABI	ankle–brachial index
AKI	acute kidney injury
ASCVD	atherosclerotic cardiovascular disease
baPWV	brachial–ankle pulse wave velocity
cIMT	carotid intima-media thickness
CAD	coronary artery disease
CKD	chronic kidney disease
eGFR	estimated glomerular filtration rate
ICU	intensive care unit
LVH	left ventricular hypertrophy
MAP	mean arterial pressure
RRI	renal resistive index
